# The difficulty with responding to policy changes for HIV and infant feeding in Malawi

**DOI:** 10.1186/1746-4358-5-11

**Published:** 2010-10-26

**Authors:** Jacqueline R Chinkonde, Johanne Sundby, Marina de Paoli, Viva C Thorsen

**Affiliations:** 1 Institute of General Practice and Community Medicine, Faculty of Medicine, University of Oslo, Norway; 2 Fafo Institute for Applied International Studies, Oslo, Norway

## Abstract

**Background:**

When and how to wean breastfed infants exposed to HIV infection has provoked extensive debate, particularly in low-income countries where safe alternatives to breastfeeding are rarely available. Although there is global consensus on optimal infant-feeding practices in the form of guidelines, practices are sub-optimal in much of sub-Saharan Africa. Policy-makers and health workers face many challenges in adapting and implementing these guidelines.

**Methods:**

This paper is based on in-depth interviews with five policy-makers and 11 providers of interventions to prevent mother-to-child transmission (PMTCT) of HIV, participant observations during clinic sessions and site visits.

**Results:**

The difficulties with adapting the global infant-feeding guidelines in Malawi have affected the provision of services. There was a lack of consensus on HIV and infant-feeding at all levels and general confusion about the 2006 guidelines, particularly those recommending continued breastfeeding after six months if replacement feeding is not acceptable, feasible, affordable, sustainable and safe. Health workers found it particularly difficult to advise women to continue breastfeeding after six months. They worried that they would lose the trust of the PMTCT clients and the population at large, and they feared that continued breastfeeding was unsafe. Optimal support for HIV-infected women was noted in programmes where health workers were multi-skilled; coordinated their efforts and had functional, multidisciplinary task forces and engaged communities. The recent 2009 recommendations are the first to support antiretroviral (ARV) use by mothers or children during breastfeeding. Besides promoting maternal health and providing protection against HIV infection in children, the new Rapid Advice has the potential to resolve the difficulties and confusion experienced by health workers in Malawi.

**Conclusions:**

The process of integrating new evidence into institutionalised actions takes time. The challenge of keeping programmes, and especially health workers, up-to-standard is a dynamic process. Effective programmes require more than basic resources. Along with up-to-date information, health workers need contextualized, easy-to-follow guidelines in order to effectively provide services. They also require supportive supervision during the processes of change. Policy-makers should ensure that consensus is carefully considered and that comprehensive perspectives are incorporated when adapting the global guidelines.

## Background

In the past two decades, there have been some difficult challenges associated with the feeding of infants born to HIV-infected mothers. Whereas breastfeeding is optimal in almost all settings, the HIV/AIDS epidemic and its potential for mother-to-child transmission of HIV (MTCT), has challenged the established notion of full breastfeeding for all. Practices such as breastfeeding, which are beneficial to growth and health, and other interventions aimed at reducing MTCT, may be juxtaposed, requiring policies to change rapidly. Recent research indicates that exclusive breastfeeding for six months carries minimal HIV risk (4%) [1], and the risk is even lower (about 1.3%), if the breastfeeding is limited to about three months [2]. Mixed feeding increases the risk of HIV transmission [3]. In low-income countries, failure to breastfeed, especially in the first year of life, exposes children to a greater risk of malnutrition and other life threatening infectious diseases [4]. The comparative risk of death from other infectious diseases, especially diarrhoea, is six times greater in formula-fed than in breastfed children in the first two months of life [5].

To prevent the risk of HIV transmission without undermining the value of breast milk for young children, the global partners (UNAIDS, WHO, UNFPA and UNICEF) developed a set of guidelines for HIV and infant feeding. The first two sets of global guidelines were issued in 1987 and 1992 and recommended continued breastfeeding by HIV-positive mothers in low-income settings [6,7]. The 1998 WHO technical consultation first introduced the option of replacements for breastfeeding. Subsequent technical consultations (2001, 2006, and recently 2009) sought to distinguish when replacement feeding, rather than breastfeeding, should or should not be chosen by HIV-positive mothers [8-11]. The guidelines generally support breastfeeding, but the difficulty lies in determining the contexts in which breastfeeding and/or replacement feeding are most appropriate. In the 2001 guidelines, assessment criteria for health workers were introduced calling for replacement of breast milk only in situations in which it was "*acceptable, feasible, affordable, sustainable and safe (AFASS)". *Otherwise, exclusive breastfeeding was recommended during the first months of life. Health workers were advised to provide specific guidance and support to the women to help them make the best infant feeding choices after receiving counselling on the risks and benefits of various options [9].

The implications of the 2001 recommendations were that HIV-infected mothers from high-income settings could avoid breastfeeding altogether because they could afford and safely practice replacement feeding. HIV transmission has been reduced to <2% in these settings as a result of antiretroviral prophylaxis for women during pregnancy and labour, elective caesarean section, and women not breastfeeding at all. In low-income settings, other prevention measures have been adopted. Governments have promoted exclusive breastfeeding followed by rapid cessation at six months because alternatives for breastfeeding have not been feasible for most mothers [12]. Ironically, empirical evidence shows that exclusive breastfeeding and formula-feeding practices are both sub-optimal in these settings [2,13]. Despite efforts to promote exclusive breastfeeding, mixed feeding continues to dominate in many sub-Saharan countries. In Malawi, although exclusive breastfeeding practices at six weeks improved from 43% to 53% between 2000 and 2004, these rates declined rapidly to 27.5% in the fourth month and 3.8% in the sixth month [14]. Similarly, in South Africa, 61% of mothers initiate breastfeeding within one hour of a child's birth. However, only 11.9% of children were still exclusively breastfed at four months, and this percentage decreased to 1.5% by six months of age [15].

According to critics, effective implementation of the 2001 guidelines in sub-Saharan Africa has failed because exclusive breastfeeding and rapid cessation are not common practices, and they are difficult to carry out in practice [16-19]. Prolonged partial breastfeeding (giving an infant some breast milk and some artificial feeds, either cow's milk, cereal, or other food) is common in these settings [20]. Rapid cessation at six months is also reported to be potentially hazardous because of the increased risk of malnutrition, infection, and infant mortality [21]. The basic principle underlying the 2001 guidelines ignores the fact that their application depends on mothers being aware of, and having access to, other practical feeding options that are commonly provided through PMTCT programmes [22]. Replacement feeding also involves the risk of inadvertent HIV disclosure as HIV-infected women are encouraged to solicit support from their close social networks [23,24] and advised to breastfeed in a different way or not breastfeed at all [19,25]. Furthermore, inadequate guidance and minimal support on the practical issues make women less likely to endure the psychological stresses of crying children as they modify their feeding practices [26].

Based on lessons from the negative outcomes of replacement feeding, a substantial body of new empirical evidence and experiences from health and infant feeding programmes, the 2001 guidelines were revised in 2006 [10]. The revised guidelines recommended that breastfeeding be continued unless cessation at six months was judged to be AFASS [10]. The difference between the 2001 and 2006 guidelines was that the former did not specify the period during which exclusive breastfeeding should be practised. It only recommended this practice in the first few months of life followed by rapid cessation. The six-month period was specified in 2006, and gradual weaning was advocated until children received a safe and adequate diet.

For policy-makers and health workers, concerns have arisen about how these recommendations should be implemented effectively. The challenge is to find ways of advising different mothers to adopt practices that will enable their HIV-exposed children to obtain optimal nutrition while minimising their HIV risk. This has been complicated in part by health workers' lack of time and skills for counselling, by the difficulty of ensuring AFASS for the women, and by confusion about the breastfeeding and weaning periods [27]. In some cases, health workers are completely bewildered by conflicting information [28].

## Methods

Our study aimed to assess policy-makers' and health workers' opinions about, and experiences with, adapting and implementing the global guidelines in Malawi. We specifically sought to explore health-facility-level support systems that were available to help HIV-infected women adhere to the infant feeding options of their choosing. Site visits to programmes that provided "better than standard" infant feeding counselling and support were also made. The purpose was to identify practices that could be replicated at the study sites and possibly in similar settings.

### Setting

We conducted this study between February 2008 and April 2009 at two internationally supported PMTCT public health facilities in Lilongwe, Malawi. Early services for PMTCT of HIV in Malawi were initiated by non-governmental agencies, but services are now available in government hospitals. The first three providers were UNICEF at Embangweni hospital in Mzimba, Médecins Sans Frontières (MSF) Luxembourg and MSF-France in the Thyolo and Chiradzulu districts, respectively. The University of North Carolina (UNC) project, which is a research institution, was the fourth provider, but was the first to collaborate with Ministry of Health (MoH) in 2002.

Two of the four UNC-supported sites were purposefully selected for this study because UNC has, and continues to provide, the bulk of PMTCT services in Malawi [29,30]. The two selected clinics varied in their settings: one semi-urban and one rural. The semi-urban clinic represented women whose partners had migrated from rural areas to town for informal employment or business. This clinic has a catchment population of over 500,000 people, provides antenatal care to nearly 60,000 pregnant women and conducts about 10,000 deliveries in Lilongwe every year. It also serves as a referral facility for high-risk maternity cases from surrounding peripheral health centres within the district. The rural clinic represented women who were engaged in agricultural activities. It has a catchment population of over 100,000 people, provides antenatal care to nearly 5,500 new pregnant women and conducts 3,000 deliveries every year. This patient load is relatively large for a rural clinic because almost 90% of Malawians live in rural areas [31].

PMTCT interventions at the UNC project, at MoH and in most programmes are provided as part of the comprehensive maternal and child health (MCH) package [32,33]. Health centres and hospitals offer discussions of general health issues in a group session for all attending women. First time attendees and those not previously tested receive a detailed PMTCT group session and group counselling, followed by individual testing. Women receive HIV testing as part of their routine antenatal care unless they specifically decline (opt-out). Government nurses and midwives provide all subsequent antenatal care, delivery and postnatal care.

Before April 2007, the UNC project provided all PMTCT programme components at four public health facilities in Lilongwe to pregnant women who had positive HIV-test results until their children reached 18 months of age. After April 2007, MoH re-organized PMTCT services in selected districts as pilot projects before a national scale-up, and this included facilities where the UNC project operated. UNC started providing PMTCT services including follow-up to HIV-infected women only up to their delivery. A national Early Infant Diagnosis (EID) programme run by MoH took over the follow-up of HIV-exposed children from six weeks after birth until their first birthday, when a final HIV-status was determined. HIV-negative children are discharged from EID and those testing positive are referred to Baylor, a paediatric HIV centre for specialised treatment and care supported by government and international partners.

### Sample, data collection procedure and analysis

Eleven purposefully selected PMTCT providers were recruited into this study. They consisted of eight nurses, two paediatricians and one lay-counsellor, who was a former PMTCT client. Research staff consisting of the first author and a male assistant initially informed all providers about the study, and those who agreed to be interviewed were invited to do so. Participants were asked about their experiences with regard to HIV and infant feeding and about the extent to which they had integrated the global guidelines when counselling women attending PMTCT services. To determine how the global guidelines were adapted to the Malawian context, five policy-makers (PM) were also interviewed. All participants were further asked about their opinions with regard to changes in the guidelines. All interviews were conducted in English, with some use of Chichewa, the local language, using semi-structured, open-ended interview guides after obtaining consent from individual participants. Each interview session lasted between 45 minutes to one hour.

To complement the interviews, the first author also conducted participant observations at the studied facilities. These observations were conducted to explore how HIV and infant feeding counselling was imparted to the women. All observations were made throughout the study period during education and group and one-on-one counselling sessions.

Toward the end of the data collection, the research staff, in collaboration with the national PMTCT advisor, identified three infant feeding programmes that the advisor classified as models of ideal care. These included one PMTCT programme at a government district hospital in central Malawi, one non-governmental facility and one Nutrition Intervention Programme in the south. Site visits to these programmes were made to observe how infant feeding counselling and support were provided to the women. Informal discussions with the respective programme coordinators were also held to complement the observations.

Interviews with all health workers were tape-recorded. Policy-makers preferred not being recorded, so notes were taken instead. Recorded interviews were then transcribed, and the handwritten notes were expanded into transcripts. The research staff independently analyzed the transcripts and identified key categories and recurrent themes, which they linked to similar sections in other interviews. Emerging themes were then jointly reviewed to reach consensus on data interpretation.

Ethical approval was granted by the Malawi National Health Sciences Research Committee and the Regional Committee for Medical and Health Research Ethics in South-eastern Norway.

## Results

Major challenges that emerged from this study were the lack of consensus about HIV and infant feeding at the national level, the lack of overarching national guidelines for health workers, weak linkages between PMTCT and other MCH services, contradictory infant feeding messages and irregular support from health workers. After describing the respondents, these emergent themes will be discussed.

### Demographic characteristics of respondents

All but one of the participants were women. With the exception of the lay-counsellor, all of the health workers had over five years of work experience in their current occupations and had received some training in HIV and infant feeding counselling. The nurses' professional training ranged from three to five years.

### Challenges at the policy level

#### *Lack of consensus among actors at the national level*

The interviews with policy-makers showed that there was a lack of consensus among actors at the national level with regard to the adaptation of the global guidelines. One policy-maker identified three government departments that dealt with HIV and infant feeding issues. These were the HIV and Nutrition Department at the Office of President and Cabinet (OPC), and the Nutrition Department, and the HIV/PMTCT units, both under MoH. She said that the HIV and Nutrition Department formulates policy, the HIV/PMTCT Unit deals with all HIV-related nutrition issues, and the Nutrition Department solely strengthens the Baby Friendly Hospital Initiative (BFHI) for all mothers, regardless of HIV status. These departments operated independently in such a way that they did not have a common understanding of HIV and the best infant feeding options for Malawi. This was reflected by the following quotes,

*"WHO infant feeding guidelines are public health policies. We know that there are internal variations among people. For those who can afford replacement feeding, let them practice that *[replacement feeding].*" *(PM 1)

*"Replacement feeding is not a choice for Malawi. There are challenges with food quality, quantity and diversity for many families because of food crises due to drought in recent years. Problems with hygiene and sanitation *[unsafe food preparation] *also risk other infections. The best remains exclusive breastfeeding." *(PM 2)

*"Replacement feeding should still be promoted in Malawi because this has worked in similar settings. Infant formula was successful in Botswana although it is difficult to measure because their programme was implemented when diarrhoea was already endemic. Thyolo district *[in Malawi] *has also registered success, only that its model is project-based *[not part of routine public services] *and as such may not be sustained after this project phases-out." *(PM 3)

This lack of proper understandings of Malawi's socio-cultural context when adapting the guidelines also partly contributed to the lack of consensus. Though one policy-maker said that MoH first evaluates the applicability of the global guidelines before adopting them, she acknowledged the inexhaustive nature of a formative study that MoH conducted prior to adopting the 2001 guidelines. In some instances, she emphasized wet nursing, which is not a common practice in Malawi. She said,

*"Malawi follows the global guidelines. We did a formative study at hospital and community levels five years ago *2003 *to determine the enabling and limiting factors of the 2001 WHO recommendations. Although the study was inexhaustive, we found that wet nursing is challenging." *(PM 2)

The absence of overarching national guidelines for health workers

The absence of overarching national guidelines made it difficult for policy-makers to develop concrete guidelines for use at the implementation level. They stated that they had encountered challenges in responding to the frequent changes at the global level, but they planned to resolve these when adopting the 2006 guidelines,

*"Malawi does not have any infant feeding guidelines for health workers. We sometimes fail to keep up with the constant changes that WHO makes on infant feeding. We are currently revising our *[Malawian] *infant and young child nutrition policy to address the new *2006 *guidelines this time with accompanying tools." *(PM 2)

*"The Ministry has not stipulated clearly what health workers should inform women about infant feeding, particularly after six months of exclusive breast feeding." *(PM 4)

With no clear guidelines for health workers in place, all participating policy-makers knew that they had subjected HIV-infected women to compromised infant feeding counselling and support, as illustrated below,

*"Our women do not often get good counselling from health workers. It is sometimes erratic and AFASS is not fully addressed." *(PM 2)

*"Health workers neglect appropriate complementary local foods during counselling*. *Even maternal nutrition is forgotten and yet it forms the backbone of child nutrition." *(PM 3)

Without adequate support for HIV-infected mothers, policy-makers were uncertain of how the 2006 guidelines would be implemented effectively in facilities that were not designated baby-friendly, as expressed in this quote:

*"Adequate support is only available in BFHI facilities. Mothers who are HIV-infected are encouraged to exclusively breastfeed their children from birth until they reach six months old, followed by an AFASS assessment. Facilities that are not Baby Friendly only encourage mothers to breastfeed and give their children appropriate replacement feeds from six months until they reach two years of age." *(PM 2)

#### Weak linkages between services

Weak linkages between PMTCT and other MCH services also raised concerns among policy-makers. They asserted that PMTCT services were being provided as vertical rather than as integrated programmes within the MCH as had originally been proposed. This in turn led to follow-up losses and missed opportunities for sustained infant feeding support,

*"There are several avenues *[opportunities] of *getting HIV-exposed children such as at under fives, family planning and postnatal clinics; conduits by which we can strengthen infant feeding counselling and support. Unfortunately, we have missed these opportunities because of weak linkages across programmes."*(PM 3)

Another said:

*"We are losing many children to follow-up through system gaps *[lack of continuity]." (PM 3)

All policy-makers acknowledged the need to go beyond education and to consider sustained counselling and support for HIV-infected mothers through a coordinated health service delivery. They further suggested promoting the use of local foods as ingredients for complementary foods because they are culturally acceptable and many families would be able to afford them.

### Challenges at the implementation level

#### Contradictory infant feeding messages

The lack of standardized information, education and communication (IEC) materials led to variations in advice on infant feeding. The biggest challenge has been finding a way to advise mothers about an appropriate weaning time. Although the advice to HIV-negative mothers was consistent (continue breastfeeding with complementary feeds until children reach two years of age), differences were noted in the advice offered to HIV-infected mothers. Most of the participating health workers indicated that they advise HIV-infected mothers to wean rapidly and introduce complementary feeds at six months. One health worker (a paediatric ART provider), however, advised HIV-infected women to continue breastfeeding their children with additional complementary feeds until they reached one year of age. She feared subjecting HIV-exposed children to malnutrition. She said that her advice was based on her previous work experience in nutrition rehabilitation units (NRU).

We do not know if her advice was based on a better or worse understanding of the empirical evidence that many mothers cannot safely stop breastfeeding at six months than that of the ten who advised mothers to stop breastfeeding at six months. Due to these inconsistencies, even at the same hospital, some health workers were frustrated as expressed below,

*"Everyone is doing what they feel is best. We use the original infant feeding policy of promoting exclusive breastfeeding followed by rapid cessation at six months. Baylor encourages breastfeeding until one year. Our messages contradict with each other. We are conveying mixed messages to the mothers. What will they follow? *[*Kaya amva ziti kaya*]*?" *[Health worker (HW) 1]

#### Irregular health worker support

Health workers employed by donor-supported agencies were observed giving practical step-by-step advice and sometimes demonstrated to the mothers how they should wean their children at six months. Conversely, MoH staff tended to give quick advice, lasting only five to six minutes, often without demonstrations. They explained that this was because they were short-staffed. The peak of the understaffing crisis was noted in December 2008 at the rural clinic when UNC turned over the provision of routine PMTCT services to MoH. Although MoH delegated some of the tasks to lay counsellors, its nurses took on a significant amount of new responsibilities for which they did not have time. In turn, PMTCT services, including infant feeding counselling, became fragmented with varying degrees of quality and negative outcomes. Infant feeding counselling, especially during subsequent antenatal visits, was rarely performed.

Concealment of HIV-positive status from partners further limited mother's support for infant feeding. Fear of negative reactions compelled them to hide their HIV-positive status as long as possible. One health worker acknowledged having observed this dilemma during home visits. She argued that it is difficult to discover this in clinics because many clients state that they have disclosed,

*"Through door-to-door counselling, we have discovered that mothers are failing to wean their children at six months because of lack of HIV-positive status disclosure. They do not have convincing reasons to tell their partners about why they need to wean their children early." *(HW 2)

This illustrates how important it is that health workers have adequate time for discussions with the women during their antenatal visits and to probe them about what is going on at home.

The sub-optimal promotion of local foods also exacerbated the challenges of implementing the global guidelines. It undermined mothers' abilities to wean their children at six months. Most health workers stated that many mothers think that milk is the only food to give to their children even after the age of six months. They ascribed this misconception to the inadequate counselling that mothers receive. They further stated that, as health workers, they lacked IEC materials to refer to when giving advice. Two asserted that the absence of IEC materials was the government's oversight,

*"We lack educational materials to give women concrete infant feeding options based on what they already have at home." *(HW 4)

*"The Ministry has not stipulated clearly on what health workers should inform mothers regarding infant feeding after six months of exclusive breastfeeding." *(HW3)

Yet another argued that:

*"The only available IEC materials on infant feeding are those that promote exclusive breast feeding until children attain six months of age. Otherwise there is none after that age." *(HW 1)

Uncertainty regarding continuity of food supplies by the World Food Programme (WFP) and other donors caused another problem. Mothers who tested HIV-positive received food aid until their children reached 18 months of age.

In another programme, children received food aid from six months until one year of age. This programme's coordinator stated that one anonymous donor brought over 200 bags of soya flour in mid-2008 that lasted until the beginning of 2009. She experienced difficulty requesting more supplies or sending reports on the flour's distribution because no one, including the district health office, knew anything about this donation. Outcomes like these contributed to weaning challenges and caused many HIV-infected mothers to stop attending their follow-up services.

### Health workers' attitudes to the changing guidelines

Appropriate advice after six months of exclusive breastfeeding was also strongly debated among most of the health workers. This is illustrated by the quotes below,

*"If a child tests negative at six months, it is good to wean them at that point. For those testing positive, breastfeeding should continue with additional feeds until they reach two years old." *(HW 3)

*"The best is to get a woman's CD4 count first. If it is high, breastfeeding should continue until the child reaches ten months old.*" (Lay counsellor)

One participant even doubted the wisdom of changing over to the 2006 guidelines,

*"If we change the guidelines now, mothers will lose trust in us. They will think that we advise them based on what goes on in our heads without considering the consequences." *(HW 4)

Instead of implicitly discouraging some mothers to continue breastfeeding after six months, health workers proposed coordinated advice at all levels to ensure sustained support for replacement feeding. They advocated for outreach services, including home visits, particularly during the transition period (from breastfeeding to not breastfeeding) and in the first three months after weaning. They also suggested that Health Surveillance Assistants (HSAs), who are the community health workers on the government payroll, or volunteers could take this responsibility.

Another suggested strengthening support for food preparation,

*"We only need to intensify infant feeding support to these mothers on how they can make what they eat *[family food] *nutritionally well *[adequate] *for their children." *(HW 5)

Yet another suggested input from PMTCT providers when revising the guidelines:

*"Why should HIV-exposed children be mixed fed *[commenting on the 2006 guidelines] *after six months of exclusive breast feeding? Is the underlying principle not the same as that of advocating condom use among HIV-infected couples? We aim at preventing further infection. The new recommendations are not good for Malawi. When making these policies, they should consult us; we can advise them better since we are the ones who deal with these *[HIV-infected] *mothers." *(HW 1)

### Best practices

Through observations at both government and NGO-supported PMTCT programmes that provided standard care, we noted that health workers were multi-skilled. At these sites, health workers provided a wide range of MCH and PMTCT services without having to refer women back and forth. Uninterrupted provision of services was observed, and this seemed to benefit both the continuity of and compliance with care. HSAs provided VCT services. Nurses gave emphasis only to specific sessions, including infant feeding counselling, during antenatal screening. There was a functioning taskforce with members from both governmental and non-governmental organizations reporting to the District Health Officer (DHO). Membership involved all programme managers responsible for PMTCT, VCT, ART, family planning, safe motherhood, laboratory, pharmacy and transport. One taskforce member stated that proceedings at their meetings included problem solving and performance assessment based on predetermined indicators.

In this district, a non-governmental organization called 'Bridge Project' was asked to help mobilize communities for male involvement in PMTCT. Through a programme called 'Bambo wa Chitsanzo' *[model man]*, the organization developed a PMTCT tool kit, which demonstrates how a child can acquire HIV during pregnancy, in-utero or during breastfeeding. The tool kit promotes exclusive breastfeeding and discourages mixed feeding. Since this NGO assumed this responsibility, the response has been overwhelmingly positive. Over 90% of mothers with children under six months of age practised exclusive breastfeeding. In March 2009, approximately 70 couples, many from the Bridge Project catchment area, accessed PMTCT services at the district hospital. This was seven times higher than the number of couples that accessed these services two months earlier.

MSF is also working in another district and has integrated all its activities within the overall government system. MSF and MoH staff jointly plan and implement PMTCT activities at both the district and health centre levels. HSAs conduct home visits and support the infant feeding practices of the women they visit. All PMTCT programme participants receive seven kgs of a supplement called Likuni Phala each month for the first six months after delivery and are encouraged to exclusively breastfeed their children. After that, the women continue to receive a reduced amount of Likuni Phala (five kgs) while their children start receiving five bottles of another nutrition supplement called "plumpy nut" each month until they reach 12 months of age.

A collaborative project between Management Sciences for Health and MoH is also implementing an action-oriented behavioural change intervention in two districts of Malawi. This project is called 'the high impact nutrition intervention for child survival'. The purpose is to reduce malnutrition among pregnant women, lactating mothers and their children by demonstrating how they can enrich their local foods. It also involves strengthening antenatal care, including PMTCT clinic attendance by pregnant women. According to the coordinator, the selected communities are those with the worst maternal and child health indicators based on the respective DHO's criteria.

Working in specific catchment areas, mother-father support groups conduct monthly home visits to households with a pregnant woman or children under age two. The purpose is to remind the families of their subsequent hospital appointments. Follow-up continues until the youngest child reaches two years of age. HIV-infected mothers receive special attention to ensure that they successfully replacement feed. For example, cooking demonstrations are held every quarter using local foods. Families are educated as to how they can enrich this food for their children's consumption. The coordinator indicated that these interventions had yielded positive results,

*"At the beginning of this project, communities used to say that they do not have any food to bring *[for demonstration]*. We however kept on encouraging them to bring anything that they had in their homes. They *[community members] *were surprised to see how their food was converted into a better meal for their children."*

## Discussion

It is not easy to advise resource-poor people on how to optimize the feeding of their infants and at the same time minimize HIV transmission risks. Malawian mothers and health workers have been exposed to HIV and infant feeding policies that have been implemented in a fragmented and uncoordinated manner and that have changed relatively often. Some of the contributing factors identified through this study were a lack of consensus about HIV and infant feeding at all levels and a general confusion about the 2006 guidelines, particularly the recommendation to continue breastfeeding after six months if replacement feeding is not AFASS.

The global partners (UNAIDS, WHO, UNFPA and UNICEF) in their 2001 HIV and infant feeding recommendations, proposed a reasonable framework within which HIV-infected women in resource-constrained countries could make their choices on the basis of their socio-economic circumstances [16]. However, policy-makers and health workers in the field had significant difficulties helping women weigh the risks and benefits of various options. This study has shown that such difficulties in Malawi relate mainly to the inability of some women to stop breastfeeding as early as the health workers want them to. Breastfeeding is the cultural norm, and infant feeding is not regarded as a mother's personal choice. HIV is also highly stigmatized, and if the mother's status becomes known, she may experience discrimination in the form of disinheritance, loss of financial support, isolation, verbal or physical abuse and gossip [24]. Mothers who do not disclose their HIV-positive status risk receiving very little or no social and emotional support. It is therefore not AFASS for mothers in this situation to stop breastfeeding at six months, even if it is economically feasible and affordable. In South Africa likewise, fear of stigmatization and disclosure has weakened the capability of HIV-positive mothers to resist norms that promote the early introduction of other foods and that question non-breastfeeding [34]. HIV-positive mothers who have tried to adhere to rapid cessation at six months have faced great distress, especially those who have not disclosed their HIV-status [26].

Whereas health workers may be considered to be in a good position to know fairly well what the mothers' lives are like and to act accordingly, they have faced several challenges. Their difficulties with, and confusion about, counselling HIV-positive mothers relate to their lack of concrete guidelines for implementation. To their credit, many have attempted to advise HIV-positive mothers, often relying on the 2001 recommendations. At the time, they lacked detailed information about the 2006 guidelines, which were current then. In view of these limitations, it is possible that they only encouraged women to stop breastfeeding at six months without necessarily helping them to weigh the risks versus benefits of the various options. Similarly, previous studies contend that the quality of counselling on infant feeding for many PMTCT programmes in sub-Saharan Africa was unsatisfactory, partly because the counsellors were not always aware of the existence of the current international guidelines [17,18,35]. Thus, while it appears logical to criticise the health workers for the confusion about guidance on HIV and infant feeding, it is crucial not to downplay the formidable role that the policy-makers have played. Their contradictory stances, which partly mirrored the different clinical aims "between those whose mandate is to prevent the spread of HIV (and who therefore would see the importance of replacing breast-feeding) and those whose mandate is child survival, who therefore promote breast-feeding as one of the pillars of child survival," [36] (p. 956), delayed the development of the overarching national guidelines.

In view of the country's high illiteracy levels, many of the women participating in PMTCT programs require significantly more time to understand the benefits and risks of exclusive breastfeeding and gradual weaning. However, human resource constraints restrict the time available for counselling even further. Malawi is one of the fifty-seven countries classified as falling below the WHO's minimum staffing recommendations in sub-Saharan Africa and Asia [37]. Nearly 50% of its posts for technical staff are vacant, and some health centres do not have a single doctor or nurse [38]. The lack of IEC materials is another reason why health workers often needed more time to counsel and support these women. With excessive caseloads and a lack of material resources, it is nearly impossible to provide women with the appropriate counselling and the follow-up they need. Paradoxically, the success of expanding PMTCT service coverage throughout Malawi [29] may come at the expense of providing adequate support and follow-up to the women.

Since PMTCT services are provided in a fragmented way and are not yet universal in Malawi [12], the absence of overarching national infant feeding guidelines has further contributed to problems with synergizing various partners' efforts. When NGOs relinquish certain services to the government for instance, their staff assume more responsibilities and are focused within the facility. This has the potential to adversely widen gaps between the needs of the facility and the community, from which most of the women's problems stem [23]. NGOs that have proven to do great work in the community do so on a small scale, and are not as interconnected as they have the potential to be. Thus, despite being available at both the facility and community-levels, efforts to support infant feeding for HIV-infected women do not provide the continuum of care that these women sorely need in order to sustain optimal practices.

The food aid that donors are again providing means a further strengthening of the efforts to promote adherence by mothers to a regimen of weaning their children early. The health workers' aspirations to make home visits for three months after weaning also have the potential to ensure that mothers do not fall back into breastfeeding again. Since the 2001 guidelines are still commonly in effect, advising mothers to wean their children early can not only do harm, especially in the context where early cessation results in infant deaths, but also result in poor communication with mothers who will continue hiding the truth about disclosure from the health workers whom they feel are not supporting their choices. The recent 2009 recommendations are the first to support the use of ARVs by mothers or their children while breastfeeding. They recommend all HIV-positive women who breastfeed and are not taking antiretroviral therapy (ART) to receive daily single-dose nevirapine for their children from birth until the end of the breastfeeding period if the mothers received zidovudine during pregnancy. Alternatively, if the women received a three-drug regimen during pregnancy, they should continue on it until the end of the breastfeeding period [11]. In addition to promoting mothers' health and providing protection against HIV infection in children, the new Rapid Advice is timely and has the potential to resolve the difficulties and confusion experienced by health workers in Malawi.

## Conclusions

The difficulty in responding to policy changes with regard to HIV and infant feeding at the national level in Malawi has affected the provision of services. Health workers have lacked guidelines and as a result, have been ineffective in counselling HIV-positive mothers. Due to the chronic human resource crisis, the time needed to provide adequate counselling simply does not exist - at this stage it is a luxury. Effective programmes seem to require more than basic resources, which of course challenges sustainability and demonstrates that good health care is not possible without real investment.

The aforementioned challenges and the confusion identified throughout this paper illustrate why the new 2009 Rapid Advice is timely and necessary. However, the process of integrating this new evidence into institutionalised actions will take time. The challenge is to find ways of keeping programmes, and especially health workers, abreast of this dynamic process. To provide services effectively, health workers need contextualized and clear guidelines that are easy to follow. They also need to be trained according to the updated instructions presented in the 2009 WHO Rapid Advice [11], and they will require supportive supervision throughout the processes of change. Policy-makers should ensure that consensus is carefully considered and that comprehensive perspectives are incorporated when adapting the global guidelines. Use of multi-skilled health workers who can provide comprehensive and coordinated support at the facility and community levels may again yield lasting solutions. In places where this has worked, a team effort has been the key. The government should take the lead, and all relevant NGOs should complement the government's efforts in a coordinated manner.

## Competing interests

The authors declare that they have no competing interests.

## Authors' contributions

JRC had primary responsibility for protocol development, data collection and analysis and writing of the manuscript. JS and MdeP participated in the protocol development and data analysis and critiqued and revised drafts of the manuscript. VCT has been involved in drafting the manuscript and revising it critically. All authors read and approved the final manuscript.
